# A Role for New Brain Magnetic Resonance Imaging Modalities in Daily Clinical Practice: Protocol of the Prediction of Cognitive Recovery After Stroke (PROCRAS) Study

**DOI:** 10.2196/resprot.9431

**Published:** 2018-05-28

**Authors:** Hugo P Aben, Yael D Reijmer, Johanna MA Visser-Meily, Jacoba M Spikman, Jeroen de Bresser, Geert Jan Biessels, Paul LM de Kort

**Affiliations:** ^1^ Elisabeth Tweesteden Hospital Department of Neurology Tilburg Netherlands; ^2^ Brain Center Rudolf Magnus Department of Neurology University Medical Center Utrecht Utrecht Netherlands; ^3^ Physical Therapy Science & Sports, Brain Center Rudolf Magnus Department of Rehabilitation University Medical Center Utrecht Utrecht Netherlands; ^4^ Department of Clinical and Experimental Neuropsychology University of Groningen Groningen Netherlands; ^5^ Department of Radiology University Medical Center Utrecht Utrecht Netherlands; ^6^ Department of Radiology Leiden University Medical Center Leiden Netherlands

**Keywords:** stroke, brain infarction, cognitive dysfunction, diffusion magnetic resonance imaging, anisotropy, diffusion tensor imaging

## Abstract

**Background:**

Cognitive impairment is common after acute ischemic stroke, affecting up to 75% of the patients. About half of the patients will show recovery, whereas the others will remain cognitively impaired or deteriorate. It is difficult to predict these different cognitive outcomes.

**Objective:**

The objective of this study is to investigate whether diffusion tensor imaging–based measures of brain connectivity predict cognitive recovery after 1 year, in addition to patient characteristics and stroke severity. A specific premise of the Prediction of Cognitive Recovery After Stroke (PROCRAS) study is that it is conducted in a daily practice setting.

**Methods:**

The PROCRAS study is a prospective, mono-center cohort study conducted in a large teaching hospital in the Netherlands. A total of 350 patients suffering from an ischemic stroke who screen positive for cognitive impairment on the Montreal Cognitive Assessment (MoCA<26) in the acute stage will undergo a 3Tesla-Magnetic Resonance Imaging (3T-MRI) with a diffusion-weighted sequence and a neuropsychological assessment. Patients will be classified as being unimpaired, as having a mild vascular cognitive disorder, or as having a major vascular cognitive disorder. One year after stroke, patients will undergo follow-up neuropsychological assessment. The primary endpoint is recovery of cognitive function 1 year after stroke in patients with a confirmed poststroke cognitive disorder. The secondary endpoint is deterioration of cognitive function in the first year after stroke.

**Results:**

The study is already ongoing for 1.5 years, and thus far, 252 patients have provided written informed consent. Final results are expected in June 2019.

**Conclusions:**

The PROCRAS study will show the additional predictive value of diffusion tensor imaging-based measures of brain connectivity for cognitive outcome at 1 year in patients with a poststroke cognitive disorder in a daily clinical practice setting.

**Registered Report Identifier:**

RR1-10.2196/9431

## Introduction

Stroke has a high global incidence of 41-316 per 100,000 persons per year [1]. Besides its physical consequences, cognitive deficits are common and occur in up to 75% of patients in the first weeks after stroke [2-5]. In half of the patients, cognition may improve [3,6]. Yet, long-term cognitive consequences of stroke include a twofold increased risk of dementia [7-9] and mild cognitive impairment [6]. Identification of those who will recover or who will have persistent cognitive impairment is important, as persistent cognitive dysfunction after stroke is independently correlated with worse long-term outcomes, such as independent living, community reintegration, and quality of life [10-14]. Moreover, recovery may be stimulated further in patients who are prone to improvement. For example, rehabilitation programs could use this information in setting realistic and attainable therapeutic goals.

Prediction of long-term cognitive outcome after ischemic stroke is, however, still inaccurate. Evidence is emerging on predictors of poor outcome, including demographic variables (eg, older age and lower level of education [2,3]). Brain imaging measures such as size and location of a stroke, the degree of white matter disease, and regional atrophy [15-21] may also determine cognitive function and long-term outcome after stroke. In contrast, fewer studies have addressed predictors of cognitive recovery among individuals with early poststroke impairment. Factors that have a positive effect on cognitive recovery are a stroke located in the right hemisphere and smoking [22,23], whereas neglect, depression, and apathy have a negative influence on cognitive recovery [23-25].

Measures of brain connectivity are of particular interest in this context. Stroke not only affects local connectivity but can also cause remote brain changes, as shown by functional MRI and diffusion tensor imaging (DTI) studies [26]. The value of brain connectivity measures has already been established for the prediction of motor recovery [27,28]. Conceivably, the relevance of measures of brain connectivity in vascular cognitive disorders (VCD) is also increasingly recognized. For example, global network efficiency assessed with DTI has been independently associated with cognitive performance in patients with cerebral small vessel disease [29-32]. Moreover, brain connectivity measures are correlated with cognition and intelligence in healthy controls [33,34] and have been suggested to provide an indication of brain resilience [34]. As such, it can be hypothesized that these measures could predict cognitive recovery after stroke. A recent study supporting this hypothesis showed that brain connectivity measures predict applied cognitive functioning 6 months after stroke [35]. Moreover, another study showed that an increased structural integrity of the contralesional hemisphere was associated with cognitive recovery after stroke [36].

The primary aim of the Prediction of Cognitive Recovery After Stroke (PROCRAS) study is to investigate whether DTI-based measures of brain connectivity predict cognitive recovery in the first year after stroke in patients with a confirmed poststroke cognitive disorder in addition to clinical, neuropsychological, and conventional imaging variables. Secondary aims are to assess the relation of DTI-based measures of brain connectivity with other outcomes. These other outcomes include deterioration of cognitive function, cognitive complaints, quality of life, participation, global health, and functional outcome. PROCRAS will also assess the feasibility of an extensive work-up including cognitive assessment and DTI in daily clinical practice.

## Methods

### Design

The PROCRAS study is a longitudinal, prospective, mono-center cohort study of cognition in patients with acute ischemic stroke, who will be followed for 1 year using 5 assessments.

### Patient Population

**Figure 1:**
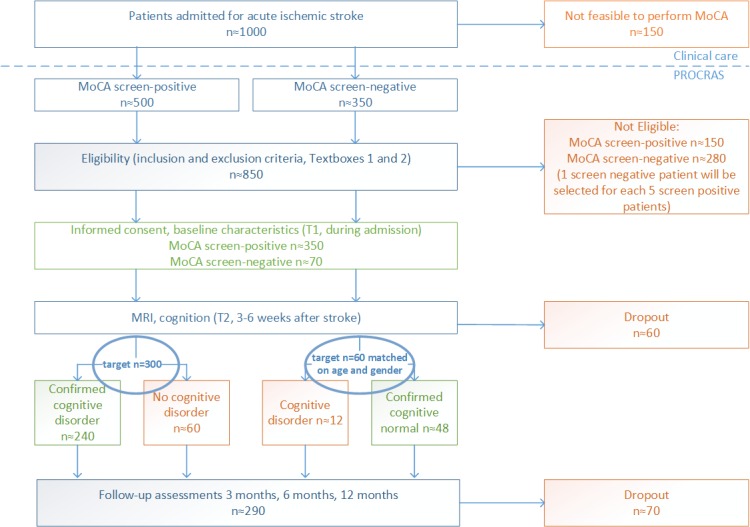
The expected patient flow in the Prediction of Cognitive Recovery After Stroke (PROCRAS) study. The target is to include 300 screen-positive patients at T2. Other numbers are estimated based on this target (MoCA: Montreal cognitive assessment; MRI: magnetic resonance imaging).

Patients will be recruited from the stroke unit of the Elisabeth-Tweesteden Hospital Tilburg, the Netherlands. This is a hospital with an admission rate of approximately 700 patients per year for the diagnosis of acute ischemic stroke. Figure 1 visualizes the anticipated patient flow; the numbers are estimated and based on unpublished pilot data and a feasibility study performed in Heidelberg [37]. In all patients admitted with a clinical diagnosis of acute ischemic stroke, aged 50 years or older, the Montreal Cognitive Assessment (MoCA) [38] is administered as part of routine care. Patients with a score below 26 (screen positive) are eligible for inclusion (Textbox 1). MoCA assessment will be reattempted if at first patients are untestable. Exclusion criteria are prestroke dementia (known diagnosis of dementia or Informant Questionnaire on Cognitive Decline in the Elderly (IQCODE≥3.6) [39]; life expectancy of less than 1 year; severe stroke expected to require long-term nursing care facilities, making patients not eligible for rehabilitation therapy; history of major neurological disease interfering with cognitive functioning; prestroke dependence in activities of daily living; insufficient command of the Dutch language; inability to participate in a neuropsychological assessment (eg, due to poor vision, severe aphasia, or if the patient is deemed untestable); and an impossibility to undergo an MRI of the brain (Textbox 2). If there is no information regarding the IQCODE, the researchers will discuss with the attending physician whether there are signs of pre-existing cognitive dysfunction. Only if there is no suspicion of prestroke dementia, patients are eligible for inclusion.

Additionally, 60 patients with a MoCA score of 26 or higher (screen negative) will be included as a reference group, matched for age and gender to the screen-positive patients (frequency matching). For this reference group, the other inclusion and exclusion criteria and study procedures are identical to the screen-positive patients.

**Inclusion criteria.**

- Clinical diagnosis of ischemic stroke
- Age ≥50 years
- Montreal Cognitive Assessment (MoCA) <26 (MoCA≥26 for the reference group)

**Exclusion criteria.**

- Prestroke dementia: Known diagnosis of dementia or Informant Questionnaire on Cognitive Decline in the Elderly (IQCODE) ≥3.6
- Life expectancy <1 year
- Severe stroke expected to require long-term nursing care facilities
- History of major neurological disease interfering with cognitive functioning
- Prestroke dependence in activities of daily living (Barthel Index<18)
- Insufficient command of the Dutch language to participate and understand questionnaires
- Impossibility to participate in a neuropsychological assessment
- An absolute contraindication to undergo an magnetic resonance imaging MRI-scan of the brain

**Figure 2:**
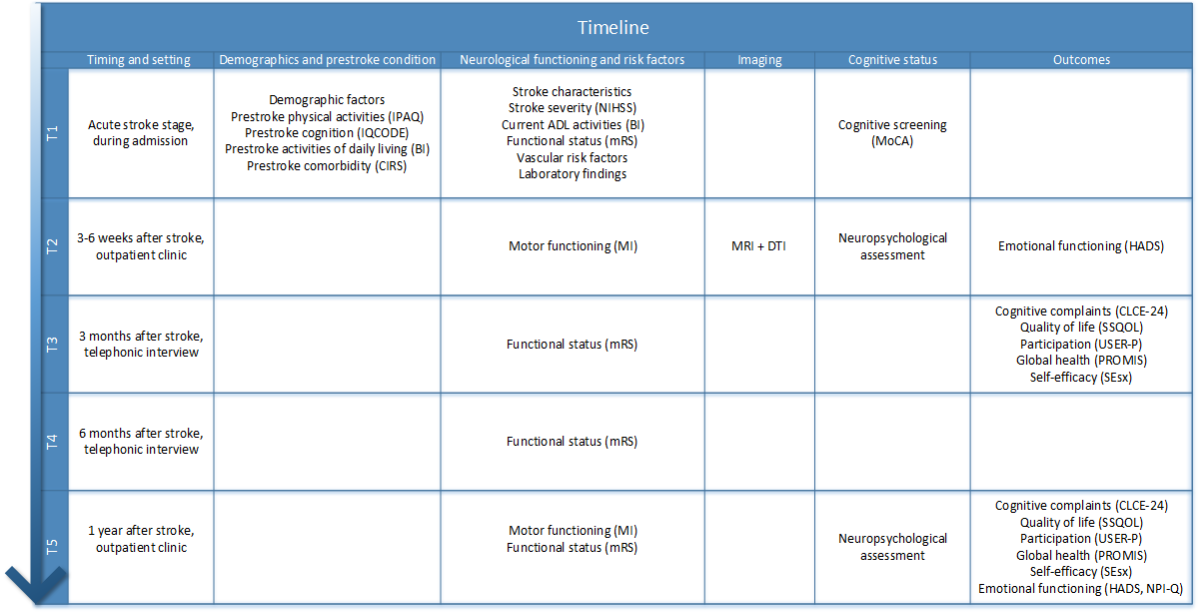
An overview of the procedures to be performed on each time point (IPAQ: the International Physical Activities Questionnaire; IQCODE: Informant Questionnaire on Cognitive Decline in the Elderly; BI: Barthel Index; CIRS: Cumulative Illness Rating Scale; NIHSS: National Institutes of Health Stroke Scale; mRS: modified Rankin Scale; MRI: Magnetic Resonance Imaging; DTI: Diffusion Tensor Imaging; MoCA: Montreal cognitive assessment; HADS: Hospital Anxiety and Depression Scale; CLCE-24: CheckList for Cognitive and Emotional problems after stroke; SSQOL: Stroke Specific Quality of Life Scale; USER-P: Utrecht Scale for Evaluation of Rehabilitation-Participation; PROMIS: Patient-Reported Outcomes Measurement Information System; SEsx: Self-Efficacy for Symptom Management Scale, NPI-Q: Neuropsychiatric Inventory Questionnaire).

It is expected that the inclusion target will be reached in 1.5 years. Patients are treated the same way as patients that do not participate in this study. This study does not interfere with treatment choices or rehabilitation therapy. To account for any confounding, it will be registered whether patients received rehabilitation therapy and what kind of therapy was provided. Moreover, basic demographic data will be collected on the source population to understand what patient population participates in the PROCRAS.

### Procedure

After informed consent, baseline characteristics will be assessed during hospital admission (T1). The second assessment takes places in an outpatient clinic 3-6 weeks after stroke (T2); the third assessment is a telephone interview 3 months after stroke (T3); the fourth assessment is another telephone interview 6 months after stroke (T4); and the fifth assessment is performed in an outpatient clinic 1 year after stroke (T5; see Figure 2). The PROCRAS study is ongoing for 18 months. From the first 100 patients at T2, 94 completed follow-up until T5.

### Measures

#### Neuropsychological Assessment

The neuropsychological assessment will be performed at T2 and T5. It is designed using the “60-minute protocol” as proposed in the vascular cognitive impairment harmonization standards [6]. Textbox 3 lists the domains and associated tests. Presence or absence of poststroke cognitive impairment is operationalized according to the criteria for VCD [40]. Patients will be classified in 3 groups:

1. Unimpaired (performance on all domains is better than 1 SD below appropriate norms)
2. Mild VCD (performance on ≥1 domains is ≥1 SD but <2 SDs below appropriate norms)
3. Major VCD (performance on ≥1 domains is ≥2 SDs below appropriate norms).

The raw score for each test will be converted into a T-score corrected for age, gender, and level of education, when possible. The score for a domain is calculated by averaging the T-scores of the tests constituting that domain. The T-score is standard score with a mean of 50 and a SD of 10. For example, a T-score of 30 translates to 2 SDs below the mean, and a T-score of 60 corresponds to 1 SD above the mean.

Only for the bells test, no normative data are available. An abnormal score on this test will be defined based on a cutoff score of a difference of 5 or more omissions between columns 1-3 and columns 5-7 [41].

#### Magnetic Resonance Imaging

MRI data will be acquired on a Philips 3 Tesla scanner (Intera, Philips, Best, the Netherlands) for each patient at T2 using a standardized scanning protocol. The protocol consists of a sagittal 3D T1-weighted, an axial T2-weighted, an axial fluid attenuated inversion recovery, an axial diffusion-weighted imaging, and a DTI sequence. Brain tissue volumes and white matter hyperintensity volumes will be automatically determined by brain segmentation, and infarct volumes will be determined by manual segmentation [42,43].

**An overview of neuropsychological tests to be performed.**

Attention and processing speed

- Reaction time test, Vienna Test System, S1, S2
- Symbol Digit Modalities Test
- Trailmaking test A

(Working) memory and learning

- Wechsler Adult Intelligence Scale (WAIS) Digit Span forward and backward
- The Rey Auditory Verbal Learning Test

Frontal-executive functions

- Controlled Oral Word Association Test
- Hayling test
- Reaction time test Vienna Test System S3
- Trailmaking test B

Language

- Boston naming test
- Semantic fluency

Visuospatial

- Bells test

Social cognition

- Facial Expressions Emotions Stimuli Test

DTI data will be analyzed and processed in ExploreDTI [44], as described earlier [45]. First, whole-brain fiber tracking will be performed. Second, the tract reconstructions are parcellated on 90 gray matter regions using the automated labeling atlas [46]. Third, a weighted connectivity matrix is obtained by scaling each present connection between each brain region by the mean fractional anisotropy of that connection. Finally, global network efficiency will be calculated by applying graph theory on the weighted connectivity matrix.

#### Other Parameters

At T1, demographic factors will be ascertained, apolipoprotein E (APOE) genotyping will be performed, stroke severity will be assessed using the National Institutes of Health Stroke Scale [47], comorbidity using the Cumulative Illness Rating Scale [48], pre-existent cognitive functioning using the IQCODE [39], pre-existent physical activities using the short form of the International Physical Activities Questionnaire [49], and pre-existent and current activities of daily living using the Barthel Index [50]. At T2 and T5, motor functioning will be assessed using the motricity index [51], and emotional functioning will be assessed using the Hospital Anxiety and Depression Scale [52] and the Neuropsychiatric Inventory Questionnaire [53].

At T3 and T5, self-efficacy will be assessed using the Self-Efficacy for Symptom Management Scale [54]; cognitive complaints using the Checklist for Cognitive and Emotional problems after stroke [55]; quality of life using the short version of the Stroke Specific Quality of Life Scale [56]; participation using the Utrecht Scale for Evaluation of Rehabilitation-Participation [57,58]; and global health using the Patient-Reported Outcomes Measurement Information System [59,60]. Functional outcome will be assessed with the modified Rankin scale [61] at T1 and T3-T5.

### Aims, Determinants, and Outcomes

#### Primary Aim

The primary aim of this study is to investigate whether DTI-based measures of brain connectivity predict cognitive recovery after 1 year, in addition to other determinants including patient characteristics and stroke severity. The main DTI marker that will be used is global network efficiency. This measure reflects the integration as well as the microstructural integrity of the white matter.

The primary outcome measure is recovery of cognitive function in the first year after stroke. This measure will be dichotomized into cognitive recovery and no cognitive recovery (ie, no change or deterioration). Cognitive recovery is operationalized as a transition from a mild VCD at 3-6 weeks (ie, T2) to no disorder at 1 year (ie, T5), or a transition from a major VCD at T2 to a mild or no VCD at T5. Hence, only patients with a confirmed VCD at T2 will be considered for this outcome. Patients that dropout will not be considered in the primary analyses. Their outcomes, obtained by telephone interview where possible, will be reported.

The secondary outcome measure is deterioration of cognitive function, operationalized as a transition from a mild VCD at T2 to a major VCD at T5 or a transition from no VCD at T2 to a mild or a major VCD at T5. For this outcome, only patients with no or a mild VCD at T2 will be considered.

In secondary analyses, we will also address change in cognitive performance as a continuous measure, using domain T-scores. For neuropsychological tests that require writing or drawing, we will take motor functioning into account as a covariate in the analysis.

#### Secondary Aims

A secondary aim is to assess the relation of DTI-based measures of brain connectivity with patient-reported outcomes and functional outcome. For this aim, the main DTI marker that will be used is, again, global network efficiency.

The outcome measures are cognitive complaints, quality of life, participation, global health, and functional outcome. The first 4 outcome measures will be assessed with questionnaires at 3 months after stroke (ie, T3) and 1 year after stroke (ie, T5). The questionnaires are repeated at T5 to assess whether a change in one of these measures occurs. Functional outcome is defined by the score on the modified Rankin Scale as assessed with a telephonic interview by a trained researcher at T1, T3, T4, and T5. All patients with completed follow-up until at least T3 will be considered for this analysis. Moreover, each of the outcome measures will be compared between the cognitive impaired group and the reference group.

Another secondary aim is to assess the feasibility of an extensive work-up including cognitive assessment and DTI in daily clinical practice.

The outcome measure will be the proportion of patients that underwent an MRI scan and neuropsychological assessment within the timeframe of 3-6 weeks at T2.

**Figure 3:**
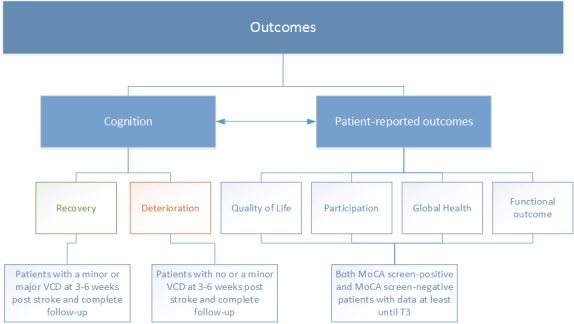
Outcomes of the Prediction of Cognitive Recovery After Stroke (PROCRAS) study and the population used in the primary analysis (VCD: vascular cognitive disorder; MoCA: Montreal Cognitive Assessment).

Figure 3 visualizes the primary and other outcomes of the PROCRAS study and the population used in the analyses.

### Sample Size Calculation

On the basis of available literature, we estimate an area under the receiver operating characteristic (ROC) curve of 0.7 for the prediction model using clinical, neuropsychological, and conventional imaging parameters, and that approximately half of the patients with a confirmed cognitive disorder will recover [3,6,62]. When adding connectivity measures to this model, 200 participants are needed to detect an increase in the area under the ROC curve of 0.1 or more, with a power of 80% and an alpha of .05. As a rule of thumb, to reliably measure the weight of a predictor, at least 10 outcome events per predictor in a multivariate model are needed. This means that a maximum of 10 predictors can be used in these models. We estimate that 30% of the patients will not complete the full study procedures until T5, and that 80% of the screen-positive patients will have a confirmed cognitive disorder at T2. Therefore, we need to obtain around 300 screen-positive patients with data at T2, to obtain 200 patients with a confirmed cognitive disorder at T2 and complete follow-up until T5. We will include 1 screen-negative patient for every 5 included screen-positive patients in the reference group.

### Statistics

First, descriptive statistics will be used. Second, to assess the additional value of DTI-based measures of brain connectivity, the 6 strongest predictors from clinical and conventional imaging parameters will be selected using univariable logistic regression with cognitive recovery as a dichotomous outcome measure. The predictive value of the multivariate model including the strongest predictors from clinical parameters and conventional imaging markers will be assessed. ROC analyses will be used to assess whether this predictive value can be improved by adding network metrics achieved from DTI. The same procedure will be performed in the prediction of the outcome measures related to the secondary aim. In the model, we will take the interval between the stroke and the assessment at T2 as covariate.

### Regulation Statement

The study will be conducted according to the principles of the Declaration of Helsinki (64th WMA General Assembly, Fortaleza, Brazil, October 2013) and in accordance with the Medical Research Involving Human Subjects Act (WMO).

### Ethics Committee Approval

The PROCRAS study was approved by the medical ethics committee of Brabant, based in Tilburg, the Netherlands. Written informed consent will be obtained from all participants.

## Results

At time of acceptance of this paper for publication, this study has been ongoing for 20 months. Thus far, 252 patients have provided written informed consent. Final results are expected in June 2019.

## Discussion

The PROCRAS study investigates whether DTI-based measures of brain connectivity predict cognitive recovery in a large stroke cohort in daily clinical practice in addition to known predictors of cognitive outcome. It adds to the existing literature because of several reasons.

First, it specifically focuses on cognitive recovery, using a selection of relevant determinants of cognitive outcome after stroke. As the mechanisms of cognitive recovery or deterioration after stroke are complex and involve multiple factors, a prediction model should incorporate each of the most important factors to provide reliable results. Moreover, by combining many of the known determinants in a large patient sample, the coherence between each of these factors can be better understood.

Second, this study combines the knowledge of different disciplines working in the stroke field, such as cognitive neuroscience, neuropsychology, and cognitive rehabilitation. Approaching VCDs from different angles may help us better understand how VCDs develop and how they continue to exist. Moreover, fundamental findings in cognitive neuroscience are not often translated in clinical practice [63]. If the diagnostic work-up proves to be feasible, results from this study can be implemented into clinical practice, benefitting individual stroke patients.

The study design has several limitations. First, the study focuses on a relatively specific patient group, because of several exclusion criteria. For example, patients that cannot undergo a neuropsychological examination due to severe aphasia are excluded. We have chosen to exclude patients with risk factors for a major VCI, as these patients have a low potential to show cognitive recovery. This would lower the sensitivity to find an effect. Second, this is a single-center study, which can affect generalizability of the results. Third, the timeframe of 3 weeks at T2 is rather broad. This might result in more patient variation, as patients show most recovery in the first weeks after stroke [64-66]. However, this timeframe of 3 weeks has been chosen for pragmatic reasons. At this point in time, information on the potential of cognitive recovery would help to make decisions in stroke rehabilitation.

In summary, the results of the PROCRAS study will support individualized PROCRAS. The results of this study may help in the psychoeducation of patients, they may add value to rehabilitation programs in setting realistic and attainable therapeutic goals, and they may help to anticipate the need for support.

## Abbreviations

DTI: diffusion tensor imaging
IQCODE: Informant Questionnaire on Cognitive Decline in the Elderly
MoCA: Montreal Cognitive Assessment
PROCRAS: Prediction of Cognitive Recovery After Stroke
VCD: vascular cognitive disorder
